# Selection and Characterization of a Novel DNA Aptamer for Label-Free Fluorescence Biosensing of Ochratoxin A

**DOI:** 10.3390/toxins6082435

**Published:** 2014-08-15

**Authors:** Maureen McKeague, Ranganathan Velu, Kayla Hill, Viola Bardóczy, Tamás Mészáros, Maria C. DeRosa

**Affiliations:** 1 Department of Bioengineering, Stanford University, 443 Via Ortega, MC 4245, Stanford, CA 94305, USA; E-Mail: mmckeagu@stanford.edu; 2 Chemistry Department, Carleton University, 1125 Colonel By Drive, Ottawa, ON K1S 5B6, Canada; E-Mails: rvelunathan@gmail.com (R.V.); khill3@connect.carleton.ca (K.H.); 3 Department of Applied Biotechnology and Food Science, Budapest University of Technology Economics, Szt. Gellért tér 4, H-1111 Budapest, Hungary; E-Mail: bardoczy@richter.hu; 4 Department of Medical Chemistry, Molecular Biology and Pathobiochemistry, Semmelweis University, Tûzoltó u. 37-47, H-1094 Budapest, Hungary; 5 MTA-BME Technical Analytical Chemistry Research Group of the Hungarian Academy of Sciences, Szent Gellért tér 4, H-1111 Budapest, Hungary

**Keywords:** aptamer, ochratoxin A, mycotoxins, *in vitro* selection, SELEX, SYBR Green I, fluorescent assay, biosensing, aptasensor

## Abstract

Nucleic acid aptamers are emerging as useful molecular recognition tools for food safety monitoring. However, practical and technical challenges limit the number and diversity of available aptamer probes that can be incorporated into novel sensing schemes. This work describes the selection of novel DNA aptamers that bind to the important food contaminant ochratoxin A (OTA). Following 15 rounds of *in vitro* selection, sequences were analyzed for OTA binding. Two of the isolated aptamers demonstrated high affinity binding and selectivity to this mycotoxin compared to similar food adulterants. These sequences, as well as a truncated aptamer (minimal sequence required for binding), were incorporated into a SYBR^®^ Green I fluorescence-based OTA biosensing scheme. This label-free detection platform is capable of rapid, selective, and sensitive OTA quantification with a limit of detection of 9 nM and linear quantification up to 100 nM.

## 1. Introduction

Ochratoxins are a group of mycotoxins produced by several fungal species of the genera *Aspergillus* and *Penicillium* [1]. Ochratoxin A (OTA), *N*-[(3*R*)-(5-chloro-8-hydroxy-3-methyl-1-oxo-7-isochromanyl)carbonyl]-l-phenylalanine (*M*_w_ = 403.81 g/mol), is the most prevalent of this group and is considered one of the most important fungal toxic secondary metabolites in food safety regulation. While it has been widely studied since its discovery in 1965 [2], public awareness of this mycotoxin has recently increased [3]. Extensive toxicity studies suggest that OTA is immunosuppressive [4], teratogenic [5], impairs blood coagulation [6], and affects glucose metabolism [7]. In particular, OTA is known for its nephrotoxicity in all animal species tested [8], and has been classified as a possible human carcinogen [9]. Several food products, such as beer, wine, nuts, liquorice, coffee, cocoa, meat, and spices, are susceptible to OTA contamination [2]. However, the occurrence of OTA in cereals, such as wheat, barley, corn, oats, and rye, is of primary concern due to the importance of these cereals in our diet. Unlike crops contaminated with other mycotoxins, OTA contamination does not result in any observable physical damage. As a result, regulatory limits (2 to 15 µg/kg for solid matrices) have been established [10] and careful monitoring and testing of food and food products is essential.

Typical OTA detection methods make use of chromatographic techniques. In particular high performance liquid chromatography-fluorescence detection (HPLC-FD) [11] has been adopted as an official method. However, significant resources and efforts have been dedicated towards modify these techniques and developing new detection methods for improved cost, speed, and detection sensitivity. Sensing platforms, such as capillary electrophoresis, solid-phase extraction–liquid chromatography–electrospray tandem mass spectrometry, and solid-phase microextraction–liquid chromatography–fluorescence detection, have been developed. Among many of these detection methods, a toxin-specific antibody is frequently applied as a molecular recognition agent [12].

Aptamers are single-stranded nucleic acid-based molecular recognition probes that can bind to a variety of target ligands with high affinity and selectivity. Selected via an *in vitro* selection procedure known as Systematic Evolution of Ligands by EXponential enrichment (SELEX) [13,14], aptamers offer several advantages over antibodies including long-term stability and low-cost of production. Additionally, aptamers can be readily modified with an assortment of reporter molecules and other chemical probes with minimal consequence to their binding properties. This has enabled their facile integration into a diverse set of biosensing and therapeutic platforms [15]. Despite the promise of aptamers as biosensors and the advantages they pose over antibodies, relatively little work has been done in aptamer-related food safety testing [16]. This is most likely due to the technical challenges implicated with aptamer selection and characterization, particularly with small molecules [17]. As a result, only a handful of aptamers exist for antibiotics, adulterants, toxic metals, and pesticides related to food [18]. Furthermore, the majority of these select aptamers have not been used for food-related testing.

One of the most promising demonstrations of food-safety related aptamers include the previously developed aptamers for the mycotoxin OTA. The first described OTA aptamer was selected using conventional SELEX strategies by Cruz-Aguado and Penner in 2008. The minimal aptamer sequence, 1.12.2, is 36 nucleotides in length and binds to OTA with a reported dissociation constant of 200 nM [19]. While only limited structural studies have been performed on this aptamer, the high guanine incidence (47%) suggests it folds into a G-quadruplex [20], a structure commonly observed with DNA aptamers [21]. In 2011, Barthelmebs *et al.* selected two additional novel OTA-binding aptamers that displayed nanomolar binding affinity to the toxin [22]. While these sequences shared two conserved sequences with aptamer 1.12.2, they were isolated under a completely different *in vitro* selection conditions.

While there are numerous challenges associated with the selection and application of small-molecule aptamers [17], the restrictions posed by sequence, structure, and binding conditions associated with each selected aptamer may cause compatibility issues with a given sensing platform of choice. Therefore, having a diverse toolset of aptamer probes to choose from for a given target may be useful for the development of novel biosensing schemes. Since practical and technical considerations limit the number of random sequences that can be applied to a selection, the sequence space is substantially unrepresented. Typically, only one of every 10^8^ possible sequences can be sampled in a typical SELEX experiment [23]. As a result, there is precedent for researchers to perform new selections on targets for which aptamers already exist, particularly using different selection conditions and strategies.

To date, the OTA aptamers have proven their utility in a variety of OTA purification and sensing technologies. For purification purposes, there are several reports demonstrating the immobilization of OTA aptamers to agarose and sepharose resins for OTA clean-up from food samples [24,25]. These aptamer-based clean-up strategies perform similarly to the commonly-used immunoaffinity columns but have an improved shelf-life. For sensing purposes, OTA aptamers have been frequently integrated into optical-based biosensors for example fluorescent [20,26,27,28,29,30], colorimetric [31,32], and luminescent [33] platforms. High sensitivity OTA detection has also been achieved with many electrochemical-based aptasensors, with detection limits as low as pg/mL [34,35,36,37,38,39,40,41,42]. Regardless of their promise, many of these methods require direct labeling with fluorescent or electrochemical probes which results in increased cost of synthesis or possible interference with aptamer folding and target binding.

Label-free strategies, such as those where there is an enhancement in the fluorescence intensity of a dye by intercalation with an aptamer, have been demonstrated several times with a variety of aptamer targets. For example, 4',6-diamidino-2-phenylindol (DAPI) [43], SYBR Green I (SG) [44], Malachite green [43,45], crystal violet [46], and OliGreen [47] have all been incorporated into aptasensors due to their ability to exhibit enhanced fluorescence after binding with aptamers. The majority of these biosensing applications exclusively use well-studied aptamers as proof-of-concept studies that bind to targets such as dyes and ATP.

In this work, we investigated the possibility of selecting novel DNA aptamers for OTA. The successful selection strategy, aptamer candidate screening, binding characterization and initial aptamer truncation is described. We then investigated whether aptamers from this selection could be successfully incorporated into a novel OTA biosensing platform utilizing SG as a DNA intercalator dye. The sensitivity range and selectivity of this assay are reported. The results suggest that this sensing scheme may be valuable in the development of low cost, portable assays for future OTA testing.

## 2. Results and Discussion

### 2.1. Selection of OTA Aptamers

Though aptamers were first isolated over two decades ago, very few have been developed that bind to small molecule targets. Practically, SELEX is a laborious, low-throughput procedure with a low success rate. Many changes have been implemented to the original selection method to improve selection success and the resulting aptamers. This has been made particularly evident in re-selection experiments that have resulted in the discovery of novel high affinity aptamers for the same targets [48]. Performing new selections and developing multiple probes for the same target also offers a unique opportunity to investigate sequence consensus motifs, structure-function relationships, and selection strategy success rates. In particular, this has yet to be explored with small molecule aptamers as their selection is much less prevalent than for proteins and cells [17].

In this work, fifteen rounds of selection were performed while gradually increasing the stringency of selection conditions. While a specific correlation between the number of selection rounds performed and the binding affinity of the selected aptamers has not been demonstrated, studies have shown that using less target-modified resin, and, therefore, less target, drastically increases the success of the selection and often results in higher affinity aptamers [21,49]. Conventional cloning and sequencing after round 15 of the OTA selections yielded several G-rich sequences. Interestingly, the previously reported OTA selections were performed with 13 and 14 iterative rounds and also resulted in G-rich sequences. Although the high contents of G are common in DNA aptamers [21,50], the consistent enrichment of G-rich sequences suggests that the target OTA may have an affinity towards G-rich motifs. Future work may investigate the evolution of aptamer libraries to determine if number of selection rounds required is a property of the target on which the selection is performed. Aptamers A08 and B08 (see Table 1) were present multiple times in the sequenced library, thus these aptamers were synthesized for further analysis. Despite the selection parallels (number of rounds and G-content present in the final library), these particular sequences did not show any sequence similarity with previously selected OTA aptamers H8 and H12 [22] or 1.12.2 [19] with the exception of a single GTGGG present in all sequences.

**Figure 1:**
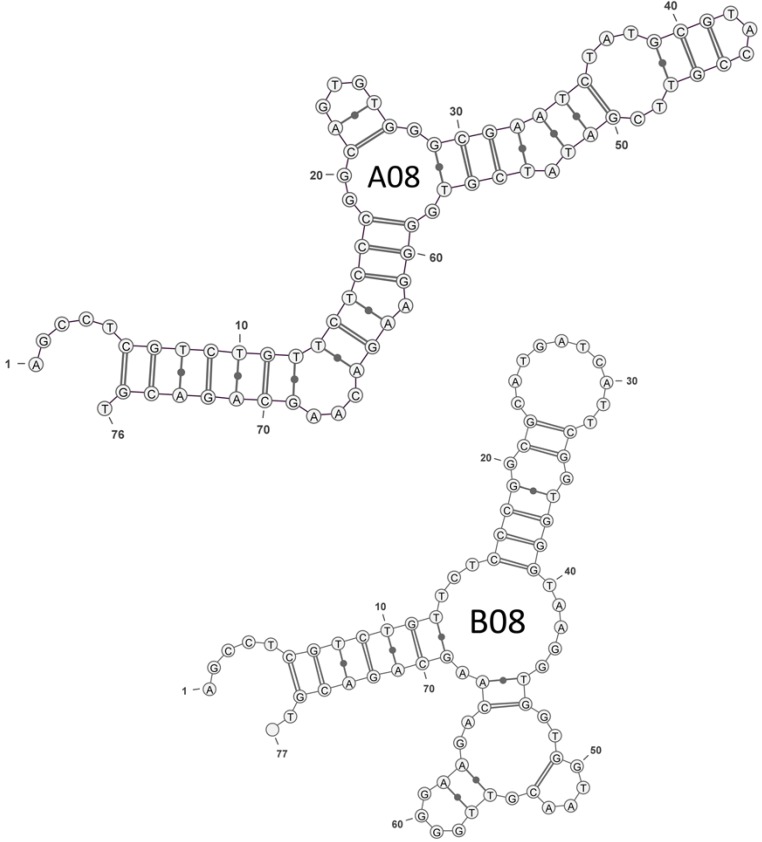
Secondary structure of OTA aptamers predicted by RNAstructure folding software [51] and rendered using VARNA software [55].

The secondary structure predictions for the full-length aptamer sequences including, the primer binding regions of A08 and B08, were generated with RNA secondary structure analysis software [51]. The structures with the lowest minimum free energy are presented in Figure 1. Each predicted structure indicates two or three stem-loop motifs commonly found with *in vitro* selected aptamers [52]. The sequence and structure of these two clones are not similar, supporting that there are several possible aptamers that bind OTA. A high incidence of guanine residues is present in both. The high incidence of guanine residues is usually characteristic of G-quadruplexes, however, G-quadruplex structures cannot be predicted using this software. Furthermore, QGRS Mapper [53] does not predict high confidence quadruplexes with these sequences. While there is some limited sequence similarity with the 1.12.2 aptamer that is predicted to fold into a G-quadruplex, our preliminary melting temperature analysis of these sequences at 295 nm did not show the characteristic signature of a G-quadruplex structure [54] (see Figure S1).

**Table 1:** The putative aptamer sequences (of the random *N*_40_ region) selected following 15 rounds of Systematic Evolution of Ligands by EXponential enrichment (SELEX). For clarity, guanine residues are italicized and the sequence motif GTGGG is underlined.

Aptamer	5'-AGCCTCGTCTGTTCTCCC-*N*_40_-GGGAAGACAAGCAGACGT-3'
A08	*GG*CA*G*T*G*T*GGG*C*G*AATCTAT*G*C*G*TACC*G*TTC*G*ATATC*G*T*G*
B08	*GG*C*G*CAT*G*ATCATTC*G**G*T*GGG*TAA*GG*T*GG*T*GG*TAAC*G*TT*G*

### 2.2. Measuring Aptamer Affinity with a Magnetic Beads-Based Isocratic Elution Assay

Measuring aptamer binding affinity (*K*_D_), determining the minimal aptamer structure, assessing target selectivity, and performing structure determination are low-throughput processes. Due to the high failure rate of small molecule aptamer selection, performing an initial screen of the selected putative aptamer clones to confirm successful binding to the target of interest is prudent. A screening step to further assess the relative target-binding properties of aptamers under various conditions is also a useful strategy for advancing the throughput of the aptamer selection workflow. We screened several aptamer candidates and binding conditions using a one-point equilibrium dialysis assay that was previously used with OTA aptamers [19]. While some information from this assay was valuable (see Supplementary Information), measured *K*_D_ values resulted in large variation (see Table S1, standard deviations between 60% and 160% from the average *K*_D_ calculated from three or more trials). As a result, better affinity methods were required to characterize these aptamers.

Affinity measurements were next attempted using an isocratic elution method with OTA-modified magnetic beads as the solid support. Typically, affinity measurements whereby the target is immobilized can be problematic as non-specific attachment to the support can confound affinity measurements [56]. A magnetic bead support was therefore chosen for this work in order to minimize this possibility, as the selections for the aptamers were performed using a sepharose resin. This strategy has been successfully applied in a similar small molecule aptamer selection [57]. Furthermore, amino groups remaining on the magnetic bead surface after OTA immobilization were capped with acetate groups to minimize non-specific electrostatic binding. 

We first validated this method for measuring aptamer affinity, using the well-characterized OTA aptamer 1.12.2 [19] (see Figure S2). This aptamer is reported to bind OTA with a *K*_D_ of 200 nM. A concentration series of 5'-labelled fluorescein aptamer was incubated with the OTA-derivatized beads. A *K*_D_ of 370 ± 250 nM was determined, consistent with previous reports.

The deviation of the affinity measurements obtained with this assay (standard deviations between 45% to 68% from the average *K*_D_ calculated using three trials) is not uncommon with this type of affinity method (see [21,56] for example) and is one of the weaknesses of this assay. This highlights one of the main challenges in small molecule aptamer development and supports the need for developing novel, rapid, and scalable methods for aptamer affinity characterization. While other reported techniques may produce more precise measurements, they are typically not broadly applicable to small molecule aptamers [17]. Other methods, such as SPR-based platforms, are scalable and accurate, but often require expensive equipment [58]. However, measuring affinity of the selected OTA aptamers with the magnetic beads assay was suitable for our needs and provided the necessary information required to incorporate these sequences into biosensing platforms.

The average *K*_D_ values from three trials, with the corresponding standard deviation values, can be found in Table 2 (binding isotherms are available as Supplementary Information, Figures S2–S4). Despite the differences in selection strategies, conditions, structure, and sequences, our newly selected A08 and B08 aptamers have binding affinities comparable to all previously selected OTA aptamers. This supports the hypothesis that aptamer binding is dependent on target features such as size and available functional groups [59]. Based on these results, both selected aptamers were further evaluated in the SG fluorescence assay.

**Table 2:** Dissociation constants for the magnetic beads assay were obtained using a simple ligand binding fit. Values are reported as the average of at least three trials +/− std. dev. Binding isotherms are available as Supplementary Information, Figures S2–S4.

Aptamer	*K*_D_ (nM)
1.12.2	370 ± 250
A08	290 ± 150
B08	110 ± 50

### 2.3. SYBR Green I Assay

#### 2.3.1. Principle of Assay

**Figure 2:**
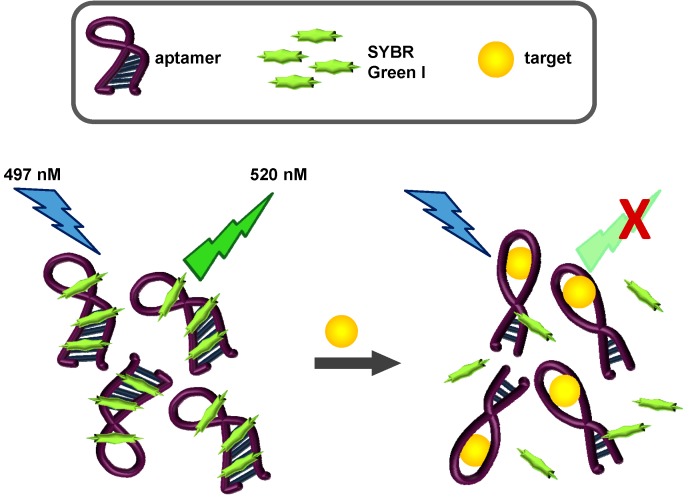
SYBR Green I fluorescence-based OTA aptasensing scheme. In the presence of the aptamer, SG fluoresces at 520 nM, due to intercalation and other hydrophobic interactions with the DNA aptamer. Increasing concentrations of the aptamer target (OTA in this assay) causes a displacement of SG, and a concentration-dependent loss of emitted fluorescence.

SYBR Green I (SG), *N*',*N*'-dimethyl-*N*-[4-[(*E*)-(3-methyl-1,3-benzothiazol-2-ylidene)methyl]-1-phenylquinolin-1-ium-2-yl]-*N*-propylpropane-1,3-diamine interacts with double stranded DNA through intercalation and binding to the minor groove [60]. Upon binding, fluorescence is significantly enhanced. SG-based assays have been used successfully for the quantification of nucleic acids [61], for real-time PCR [62], in flow cytometry [63], and imaging [64]. More recently, the fluorescent properties of SG and the binding properties of aptamers have been coupled to develop label-free assays. In these proof-of-concept designs, the thrombin and ATP aptamers were employed [44,65,66,67]. Incubation of these aptamers with SG, as expected, resulted in enhanced emission of green light upon excitation with blue light. When the cognate targets of each aptamer were incubated with the aptamer-SG complexes, the target preferentially bound to the aptamer, competing for SG binding positions. As a result, SG emission decreased. We tested whether our newly selected aptamers would be suitable for this strategy and consequently allow label-free biosensing of OTA. A schematic of our OTA label-free sensing scheme is shown in Figure 2.

#### 2.3.2. Biosensing of OTA

**Figure 3:**
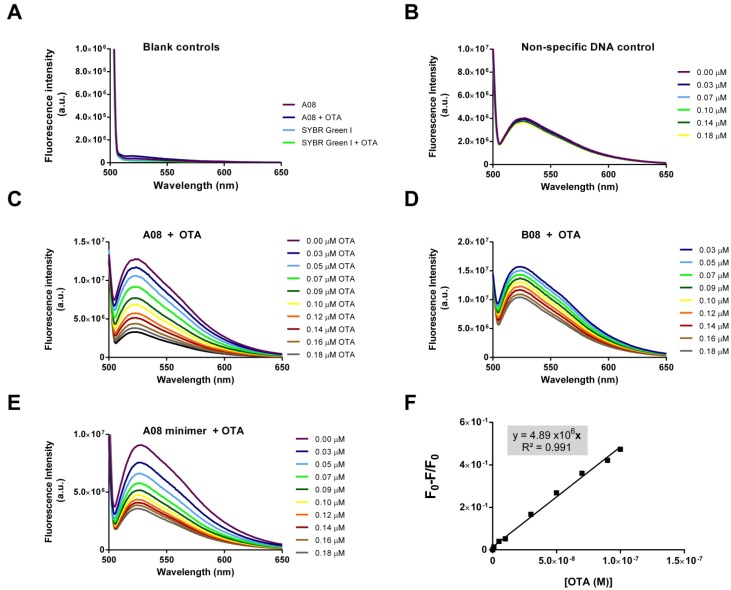
The SG-based label-free assay (**A**) Aptamer A08, OTA, SG, and SG with OTA show no fluorescence from 500–600 nm; (**B**) Combining DNA and SG results in significant emission at 520 nm. A non-specific DNA control does not respond to the addition of OTA; (**C**–**E**) The A08, B08 and A08 minimer aptamers selected in this experiment display enhanced fluorescence when incubated with SG. Upon the addition of OTA, a concentration dependent loss of fluorescence is observed; (**F**) The A08 minimer-based sensor has a linear response between 9 and 100 nM (*y* = 4.89 × 10^6^*x*; *R*^2^ = 0.991).

Figure 3 shows the results of the SG-based assay. OTA, SG, and DNA alone do not contribute to any fluorescence signal from 500 to 600 nm. Furthermore, combining OTA and SG or OTA and aptamer does not result in any measureable signal (panel A). Upon excitation at 497 nm, maximal fluorescence emission occurs only when DNA and SG are combined (panel B). Non-specific control DNA that displays no binding to OTA shows no response with increased concentrations of OTA (panel B). However, when the validated OTA-binding aptamers are used in the assay, a concentration dependent decrease in fluorescence is observed with the addition of incremental concentrations of OTA. 

Both aptamers A08 and B08 demonstrated this concentration dependent decreases; however, A08 resulted in a greater response to OTA (panel C and D). Therefore, we also tested a truncated version of the A08 aptamer (primer binding regions removed) in an effort to reduce the cost of the sensor. This A08- mini aptamer performed similarly to its full-length parent (panel E).

When the fluorescent signal is normalized from the blank (*i.e.*, no target added), a concentration dependent linear relationship can be plotted. The normalized linear plot for the minimal aptamer A08 with OTA is displayed in Figure 3 (panel F). The biosensor performance was assessed with the normalized fluorescence signal, which was determined at 520 nm using Equation 1 (in Experimental Section). The A08 minimer can thus be used to detect OTA up to 100 nM, with a limit of detection (LOD) [68] of 9 nM (3.6 ppb).

**Figure 4:**
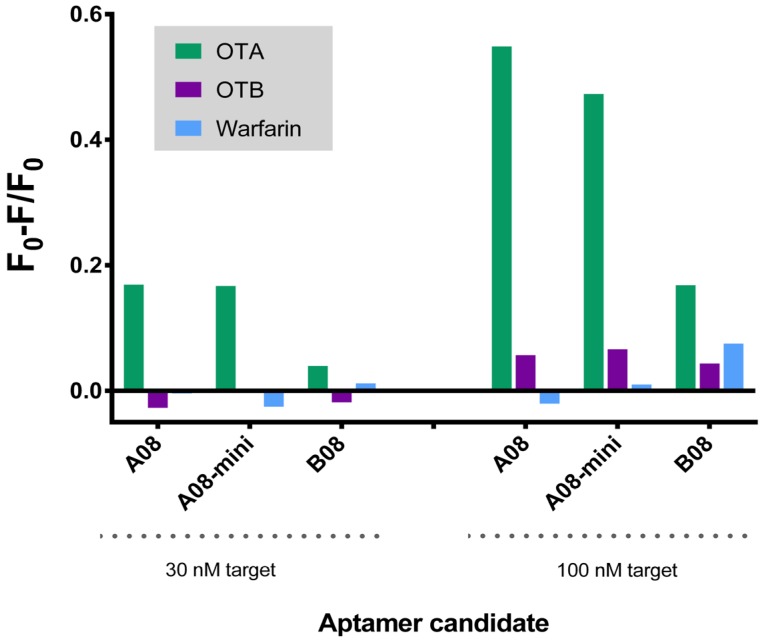
Selectivity of the SG aptamer biosensor at 30 and 100 nM concentrations of targets. The sensor is more sensitive to the target OTA compared to similar compounds. All the fluorescence spectra for the aptamers with these three targets are available as Supplementary Information (Figures S5–S7).

We next assessed the selectivity of this assay against similar toxins. While the selected aptamers display high affinity for OTA, previous reports have demonstrated that affinity and selectivity are not necessarily related [69]. All aptamers were tested with OTB and warfarin. OTB in particular, differs from OTA by only a single chloro group. All aptamers displayed preferred recognition towards OTA at both low (30 nM) and high (100 nM) concentrations of target. However, B08 resulted in only modest selective detection of OTA (2–3 fold signal compared to OTB and warfarin). Both A08 and its minimer resulted in 6–10 fold enhanced signal of OTA compared to OTB and 30–40 fold improvement compared to warfarin (Figure 4). Comparing the structures of these compounds, it is not surprising that this biosensor shows modest response to OTB but negligible response to warfarin. OTB differs from OTA by a single chloro group but warfarin replaces the phenylalanine group of OTA with a simple phenyl. While antibodies may not necessarily distinguish between these subtle differences in structure, there are numerous examples of aptamers being able to recognize such variation. The selectivity trend we observed is very similar to that found with the original OTA aptamer [19].

Using the magnetic beads assay, the B08 aptamer sequence displayed higher affinity towards OTA compared to A08, however, B08 performed relatively poorly in the SG biosensor platform. One possibility is that this particular aptamer is not well suited for this sensing scheme. The B08 binding kinetics may not be sufficient to compete with SG. Alternatively, the OTA binding pocket may not undergo conformational change, and, thus, does not affect SG intercalation and binding. The other possibility is that B08 interacts non-specifically with the magnetic beads or prefers a linker-modified OTA molecule.

The SG-based biosensor platform presents several advantages compared to other mycotoxin detection methods. This assay was rapid, quantitative, inexpensive, and label-free. In particular, the implementation of a DNA sensor component as opposed to antibody-based detection, reduces the costs, and improves the shelf-life of this assay compared to most current mycotoxin clean-up and analytical methods. Development of this assay was an important step towards the realization of inexpensive, robust, and innovative detection technology and may be the solution to the limitations of the existing mycotoxin detection techniques. 

## 3. Experimental Section

### 3.1. Materials

Buffers were prepared with Millipore Milli-Q deionized water at 18 MΩ. Buffering agents, salts, *N*-(3-Dimethylaminopropyl)-*N*'-ethylcarbodiimide hydrochloride (EDC), OTA from Petromyces albertensis ≥98% (TLC), warfarin, and SG (10,000×) were purchased from Sigma-Aldrich (St. Louis, MO, USA). OTB was purchased from Santa Cruz Biotechnology (Dallas, TX, USA). All molecular biology grade electrophoresis chemicals were purchased from BioShop Canada (Burlington, ON, Canada). EAH sepharose 4B was obtained from GE-Healthcare (Uppsala, Sweden). The streptavidin coated high-binding capacity plate was purchased from Thermo Fisher Scientific (Rockford, IL, USA). The DNA Taq polymerase, PCR components, and Dynabeads^®^ M-270 Amine were purchased from Life Technologies (Carlsbad, CA, USA). A p-GEM-T-easy vector by T/A cloning kit was received from Promega (Madison, WI, USA). Phosphoramidites, modifiers, activator, deblock, capping, and oxidizing reagents were obtained from Glen Research (Sterling, VA, USA). Standard support columns and acetonitrile were purchased from BioAutomation (Plano, TX, USA). Ultra High Purity 5.0 argon was purchased from Praxair Canada (Mississauga, ON, Canada). Sulfo-NHS acetate, Amicon-Ultra 0.5 mL 3 kDa centrifuge units were purchased from Fisher Scientific Canada (Ottawa, ON, Canada). UV/Vis absorption spectra were obtained using a Cary 300 Bio UV-Visible spectrophotometer (Varian, Palo Alto, CA, USA) and fluorescence was measured with Fluorolog Fluorescence Spectrophotometer with a SpectrAcq controller (Horiba Jobin Yvon, Edison, NJ, USA).

### 3.2. OTA SELEX Experiments

#### 3.2.1. Preparation of OTA Resin

OTA was coupled to EAH Sepharose 4B according to manufacturer’s protocol. Briefly, a 5 mM solution of OTA in deionized water was prepared and adjusted to a pH of 4.5. The OTA solution was mixed with sepharose at a 1:1 *v*/*v* ratio. 0.1 M of EDC was prepared in water with the pH adjusted to 4.5 and added to the OTA and sepharose mixture. The reaction was incubated at room temperature for 4 h with gentle shaking. After the first three hours, the pH was again adjusted to 4.5. After the incubation period, the gel matrix was washed with three cycles of alternating washes consisting of 0.1 M acetate buffer pH 4.0 containing 0.5 M NaCl and 0.1 M Tris-HCl buffer pH 8 containing 0.5 M NaCl. The OTA resin was stored at 4 °C until use.

#### 3.2.2. SELEX Library

The oligonucleotide library was synthesized at 1 µmol scale and purified using RP-HPLC (Metabion, Martinsried, Germany). This library was composed of 40 random nucleotides flanked by two fixed regions. 5'-AGCCTCGTCTGTTCTCCC-*N*_40_-GGGAAGACAAGCAGACGT-3'. The forward primer, SELF, 5'-AGCCTCGTCTGTTCTCCC-3', and reverse primer, SELR, 5'-BIOTIN-ACGTCTGCTTGTCTTCCC-3', were also synthesized and prepared for the selection procedure.

#### 3.2.3. *In Vitro* Selection

SELEX was performed as described by Murphy *et al*. with a few modifications [70]. The SELEX library (1 nmol) was dissolved in and 0.05% Triton-X and selection buffer (SB) containing 10 mM Na_2_HPO_4_, 2 mM KH_2_PO_4_, 2.7 mM KCl, 137 mM NaCl, pH 7.4. This library was incubated with 10 µL of the prepared OTA resin at room temperature for one hour. The resin was washed three times with the SB to eliminate non-specifically bound sequences. The putative aptamers were then eluted using 10 µL of 50 mM NaOH and purified using a phosphate buffered saline (PBS)-equilibrated desalting column then transferred directly to the PCR reaction. 

The 100 µL PCR reaction mixture contained 10 µL of 10× reaction buffer, 1.5 units of Taq polymerase, 1 µM of the two primers (SELF and biotinylated SELR), 0.8 mM of each deoxyribonucleotides (dNTPs) and 1.5 mM MgCl_2_. Amplification was performed by heating to 95 °C for two minutes followed by 18 cycles of 30 s at 95 °C, 30 s at 56 °C and 30 s at 68 °C, followed by a final extension at 68 °C for two minutes. After checking the PCR product quality on 2.5% agarose gel, 23 µL of 5 M NaCl was added to the remaining 90 µL PCR mixture. This solution was divided into two parts and incubated with a Reacti-Bind streptavidin coated high-binding capacity plate and washed three times with 1 mL of PBS-Tween. The nonbiotinylated strands were separated from the immobilized complementary strand by incubating with a 30 µL of fresh 100 mM NaOH for five minutes. The ssDNA was removed, diluted using SB, pH adjusted, and heated to 95 °C for five minutes and immediately placed on ice until the next round of SELEX.

10 µL of the OTA resin was used in cycles one through seven. To increase the stringency of selections, only 5 µL of OTA resin was used in cycles 8 through 11, and 2.5 µL in cycles 12 through 15. Negative selections were performed using unmodified EAH Sephadex 4B prior to cycles 4, 8, and 12. Following cycle 15, the final PCR of the selected ssDNA sequences was performed using the SELF and nonbiotinylated SELR primers. A fraction (4 µL) of this PCR mixture was ligated into p-GEM-T-easy vector by T/A cloning and transformed into DH5α *Escherichia coli* competent cells. Twenty-one colonies were selected and tested for the presence of the correct inserts using colony PCR with M13 forward and reverse primers. The PCR products were purified by polyethylene glycol precipitation and sequenced using the M13 promoter primer [71]. 

### 3.3. Oligonucleotides

The following table lists the DNA sequences used in the aptamer characterization and biosensor assays.

### 3.4. Characterization of OTA Aptamers

#### 3.4.1. Aptamer Synthesis

Aptamer sequences A08, B08, A08 minimer, 1.12.2, and the non-specific control DNA (see Table 3) were synthesized unmodified or 5'-fluorescein labelled (6-FAM, Glen Research, Sterling, VA, USA) using standard phosphoramidite chemistry on a MerMade 6 (Bioautomation, Plano, TX, USA). DNA was purified with a denaturing polyacrylamide gel electrophoresis (12%) followed by clean-up with Amicon YM-3 Centrifugal Filter Devices (Fisher Scientific Canada, Ottawa, Canada). Sequence synthesis was verified through molecular weight verification using electrospray ionization (ESI) mass spectrometry (Novatia LLC, Monmouth Junction, NJ, USA).

**Table 3:** DNA oligonucleotides used in the aptamer characterization and biosensor study.

Aptamer	Sequence 5' to 3'
A08	AGCCTCGTCTGTTCTCCCGGCAGTGTGGGCGAATCTATGCGTACCGTTCGATATCGTGGGGAAGACAAGCAGACGT
B08	AGCCTCGTCTGTTCTCCCGGCGCATGATCATTCGGTGGGTAAGGTGGTGGTAACGTTGGGGAAGACAAGCAGACGT
A08 minimer	GGCAGTGTGGGCGAATCTATGCGTACCGTTCGATATCGTG
1.12.2	GATCGGGTGTGGGTGGCGTAAAGGGAGCATCGGACA
Non-specific control	AGCACGTTGGTAGGTCGGTTTGGGTTTCGTGC

#### 3.4.2. Magnetic Beads Binding Assay

##### 3.4.2.1. Conjugating OTA to Magnetic Beads

1 mL aliquots of amino-modified magnetic beads were magnetically separated using a DynaMag-2 magnet with approximately 10 min of incubation before the supernatant was removed and discarded. The beads were then washed three times using 0.1 M MES buffer (pH 4.5). Freshly prepared 1 M EDC (0.5 mL) and 1 mL of 60 µM OTA were added to the beads and incubated for 2 h at room temperature. The solution was magnetically separated from the beads and the amount of unbound OTA was measured using fluorescence to confirm successful conjugation. The beads were then washed twice with 0.1 M sodium bicarbonate buffer (pH 8.5). A 1 mL aliquot of freshly-prepared solution of sulfo-NHS acetate (0.05 M) was then reacted with the beads for 2 h to block any unreacted amine groups. Before storage, the beads were washed three times with 1.0 M tris buffer (pH 7.4) and three times with selection buffer. 

##### 3.4.2.2. Determining Dissociation Constants with a Magnetic Beads-Based Isocratic Elution Method

A series of 5' fluorescein modified aptamer concentrations ranging from 8 nM to 1 µM were heated to 90 °C for five minutes then cooled on ice. 90uL aliquots of OTA derivatized beads were separated on the DynaMag-2 and washed three times with SB. 90 µL of each aptamer dilution were incubated with the beads for 1 h. The supernatant was discarded and unbound aptamer was removed with four washes with SB. Bound DNA was then eluted with SB and heating at 90 °C for ten minutes. The supernatant was collected and filtered to remove any remaining beads that often impair the fluorescence background measurements. The elution step was repeated to ensure complete recovery of bound DNA and pooled. The fluorescence of eluted DNA for each aptamer dilution was measured (excitation λ = 490 nm; emission λ = 520 nm) using the Fluorolog Fluorescence Spectrophotometer (Horiba Jobin Yvon, Edison, NJ, USA). The average fluorescence was plotted against aptamer concentration in Sigma Plot (Version 12.5; Systat, San Jose, CA, USA) and fit using a simple ligand binding equation to determine the *K*_D_. The experiment was performed three times. 

### 3.5. Biosensing of OTA with the SG Assay

Each aptamer, SG and OTA dilutions were prepared in SB. First, aptamer was heated to 90 °C for five minutes and cooled to room temperature before use. 4 µL of SG (1×) and 4 µL of aptamer (10 µM) were mixed together. A range of OTA, OTB or warfarin concentrations varying from 0 to 0.18 µM were prepared in SB and added directly to the SG-aptamer mixture to a final volume of 125 µL. The fluorescence emission spectra were recorded from 500 to 650 nm using an excitation wavelength of 497 nm. The fluorescence at 520 nm was used to calculate the biosensor signal using Equation (1), where *F*_θ_ is the fluorescence intensity in the absence of OTA and *F* is the fluorescence intensity at a given concentration of OTA.


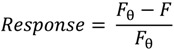
(1)

## 4. Conclusions

A new selection, using slightly more stringent conditions, was performed against OTA to isolate improved binding motifs for this target. Several unique sequences were elucidated from this selection revealing one short consensus sequence and a clear relationship between G-rich motifs and OTA affinity. Characterization revealed that the sequences bind to OTA with an apparent *K*_D_ in the nanomolar range, displaying similar binding properties with all other reported OTA aptamers. Finally, the aptamers were successfully incorporated into the first SG-based biosensing platform allowing label-free OTA quantification. This method was efficient, sensitive and displayed excellent selectivity against OTB and warfarin. This scheme may be exploited in the development of portable assays, which would allow rapid OTA detection in food samples directly in the field.

## Supplementary Files

Supplementary File 1
