# Supplementary material for: Selection and Characterization of a Novel DNA Aptamer for Label-Free Fluorescence Biosensing of Ochratoxin A
Source: Toxins (Basel). 2014 Aug 15;6(8):2435–52. doi: 10.3390/toxins6082435 (PMC4147592; doi:10.3390/toxins6082435)
Supplement: Supplementary File 1 [file toxins-06-02435-s001.pdf]

Supplementary Information

Figure S1. Sample DNA denaturation profiles for aptamers A08 and B08 at 295 nm using a ramp rate of 0.20 °C/min and 5 °C/min, respectively. Hypochromicity, which is represented by a decrease in absorbance, is not observed thus suggesting an absence of G-quadruplex formation.

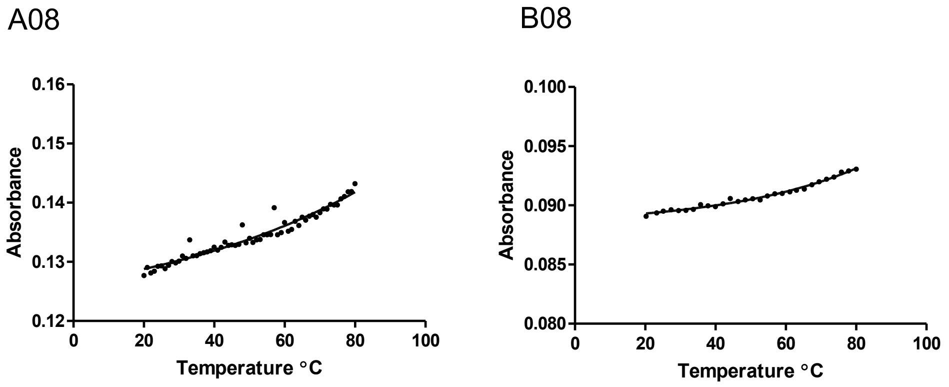

Measuring Aptamer Binding with Equilibrium Dialysis

A one-point equilibrium dialysis assay was previously used with OTA aptamers to measure the select aptamer binding affinity[19]. Equilibrium dialysis has the advantage of measuring binding directly in solution. Therefore, this assay was used to screen the newly selected aptamers and their binding conditions. In this assay, the aptamer candidates and OTA are incubated in a loading chamber of the dialysis tube. OTA can diffuse across the membrane (MWCO = 10,000 g/mol) while the larger aptamer remains in the original loading chamber. The presence of a high affinity aptamer limits the diffusion of OTA into the receiving chamber. The fraction of OTA in each chamber (Equation (S1), Supplementary Experimental) can be measured and used to calculate K_D (Equation (S2), Supplementary Experimental) [19].

Screening with this one-point assay resulted in large variation in K_D (standard deviations between 60% and 160% from the average K_D calculated from three or more trials). Notwithstanding, both sequences displayed OTA binding and A08 consistently demonstrated higher affinity (K_D 0.47 ± 0.32 µM). Using the more promising A08 aptamer, we next sought to screen optimal aptamer binding conditions, in particular including solubilizing agents that may be useful in improving OTA solubility for testing food samples (see Table S1).

Table S1. Affinity of the A08 aptamer with OTA using various buffer conditions. Dissociation constants for the equilibrium dialysis method were calculated from Equations (1) and (2).

Buffer conditions	K_D (μM)
Selection buffer (SB)	0.47 ± 0.32
SB + 2.5 % DMSO	18 ± 28
SB + 0.05 % Triton-X100	9 ± 6
SB (HEPES) + 20 mM Ca^{2+}	0.86 ±0.55

These solubilizing agents did not improve binding. Despite the significant error in these measurements, these compounds appear to reduce aptamer binding. However, it is possible that Triton X-100 allows the formation of bubbles in the dialysis chambers which could affect the approach to equilibrium.

Finally, previous reports demonstrate up to a 7-fold improvement in binding to OTA with the addition of calcium [19]. Note that 10 mM HEPES was substituted for the sodium phosphate buffer for this assessment due to the formation of insoluble calcium phosphate compounds. No improvement in binding was observed with the addition of up to 20 mM Ca^{2+} (K_D = 0.86 ± 0.55 μM).

While the equilibrium dialysis assay is low-cost and simple, the affinity measurements resulted in significant error. Furthermore, for accurate K_D determination, a full saturated binding curve is required [56]. Valuable information was obtained with this analysis; however, alternative methods are needed to confirm the binding characteristics of the selected aptamers. Another disadvantage of this assay is that it cannot be broadly applied to all aptamer-target pairs. For example, the appropriate molecular weight cut-off for these devices cannot always be purchased for the aptamer-target system and often the small molecule targets non-specifically interact with the membranes themselves.

Figure S2. Binding isotherm for aptamer 1.12.2 with OTA obtained using the magnetic beads-based isocratic elution assay. The curve was generated using Sigma Plot (Version 12.5). The K_D was determined to be 370 ± 250 nM.

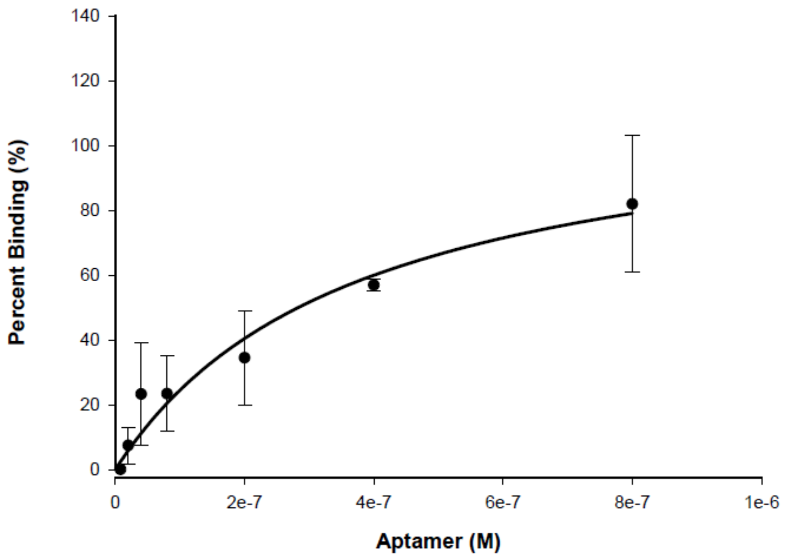

Figure S3. Binding elution assay. The curve was generated using Sigma Plot (Version 12.5). The K_D was determined to be 290 isotherm for aptamer A08 with OTA obtained using the magnetic beads-based isocratic ±150 nM.

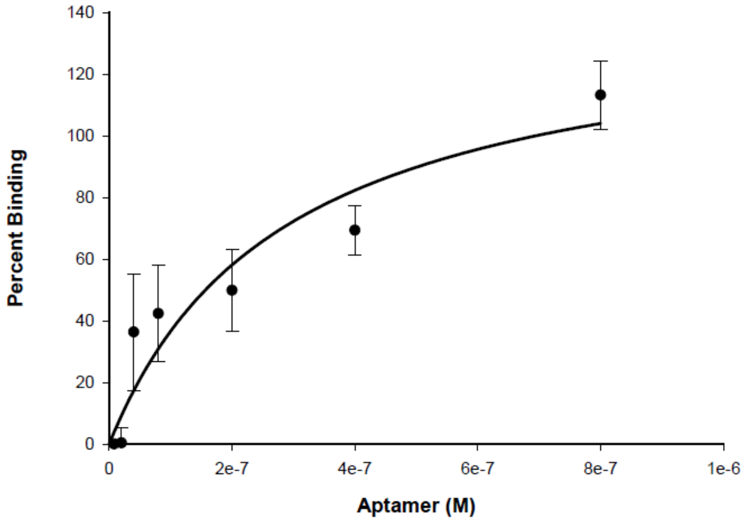

Figure S4. Binding isotherm for aptamer B08 with OTA obtained using the magnetic beads-based isocratic elution assay. The curve was generated using Sigma Plot (Version 12.5). The K_D was determined to be 110 ± 50 nM.

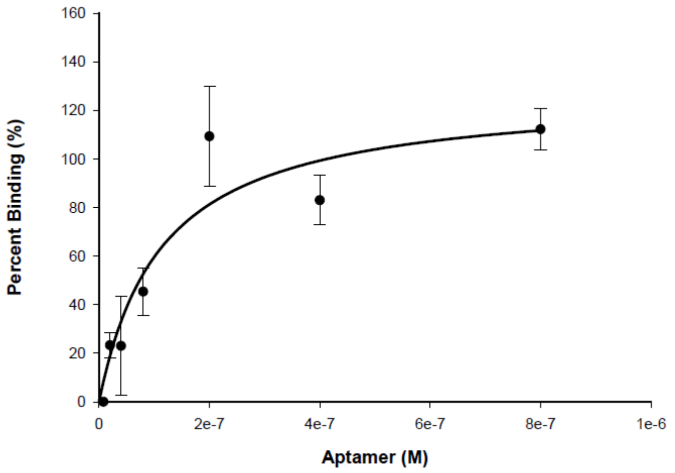

Figure S5. Fluorescence spectra (excitation 497 nm) from SG and A08 aptamer and various concentrations of OTA, OTB and warfarin. A concentration dependent decrease in fluorescence occurs with an increasing concentration of OTA; however, very little change in observed upon the addition of OTB and warfarin.

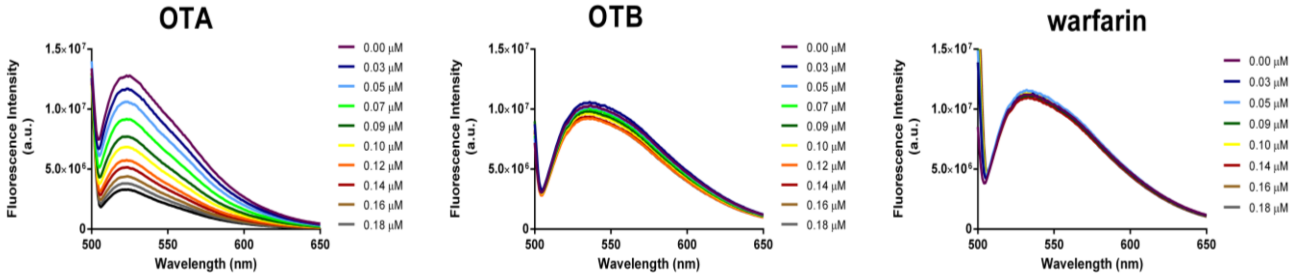

Figure S6. Fluorescence spectra (excitation 497 nm) from SG and B08 aptamer and various concentrations of OTA, OTB and warfarin. A concentration dependent decrease in fluorescence occurs with an increasing concentration of OTA; however, very little change in observed upon the addition of OTB and warfarin.

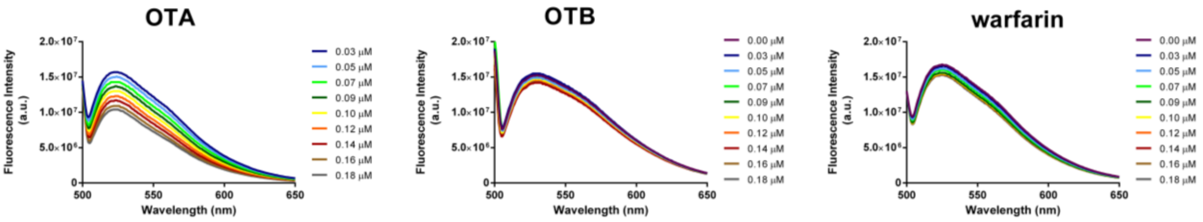

Figure S7. Fluorescence spectra (excitation 497 nm) from SG and A08 minimer aptamer and various concentrations of OTA, OTB and warfarin. A concentration dependent decrease in fluorescence occurs with an increasing concentration of OTA; however, very little change in observed upon the addition of OTB and warfarin.

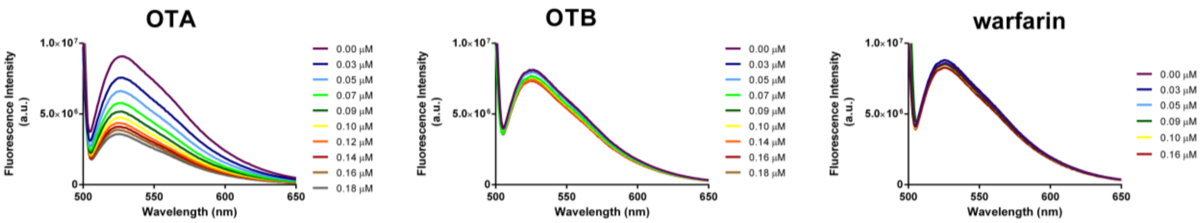

Supplementary Experimental

Thermal Denaturation Studies

The DNA aptamer candidates (2 μM) were prepared in buffer and transferred into a 10 mm open top quartz cuvette. A Varian Cary 300 Bio UV-Vis spectrometer with a 6x6 Peltier-Thermostatted Multicell Holder was set to heat the samples from 20 °C to 80 °C while measuring the absorbance at 295 nm at a ramp rate of 5 °C/min. This was repeated at a ramp rate of 0.20 °C/min. The experiment was also performed by pre-mixing the aptamers with OTA in a 1:5 (DNA:target) ratio. The absorbance values measured were plotted against temperature using GraphPad Prism 5.

Equilibrium Dialysis

The procedure was adapted from Cruz-Aguado *et al.* [19]. Single-use Sample Dispo Equilibrium Dialysis units with a 10,000 g/mol molecular weight cut-off were used. First, to ensure the aptamer sequences were unable to pass through the semi-permeable membrane, 75 μL of a 20 μM solution of aptamer AO8 in SB was added to the loading chamber (A_0). After the 48-hour incubation time, the concentration of DNA in the receiving chamber was measured using UV-Vis. Unmodified aptamer sequences were dissolved in appropriate buffer at 20 μM and mixed with 200 nM OTA. 75 μL of this solution was loaded into the loading chambers of separate dialysis units with 75 μL of the corresponding buffer solution in the receiving chamber. The units were allowed to gently shake at 60 rpm horizontally for 48 h to permit equilibration between the chambers. Following incubation,

fluorescence measurements of the loading (F_l) and receiving (F_r) chambers were obtained by direct fluorescent measurement (excitation λ = 375 nm; emission λ = 430 nm) using the Fluorolog Fluorescence Spectrophotometer. The aptamer affinity was calculated using Equations (S1) and (S2).

The fraction of bound OTA (f) was determined as:

$$f = \frac{F_l - F_r}{F_l} \tag{S1}$$

The dissociation constant (K_D) was estimated as:

$$K_D = \frac{[A_0]}{f} - [A_0] \tag{S2}$$
